# Supplementary material for: Increased Circulating Cell-Free DNA in Eosinophilic Granulomatosis With Polyangiitis: Implications for Eosinophil Extracellular Traps and Immunothrombosis
Source: Front Immunol. 2022 Jan 12;12:801897. doi: 10.3389/fimmu.2021.801897 (PMC8790570; doi:10.3389/fimmu.2021.801897)
Supplement: Supplementary file 1 [file DataSheet_1.docx]

Supplementary Material

# Supplementary Figures


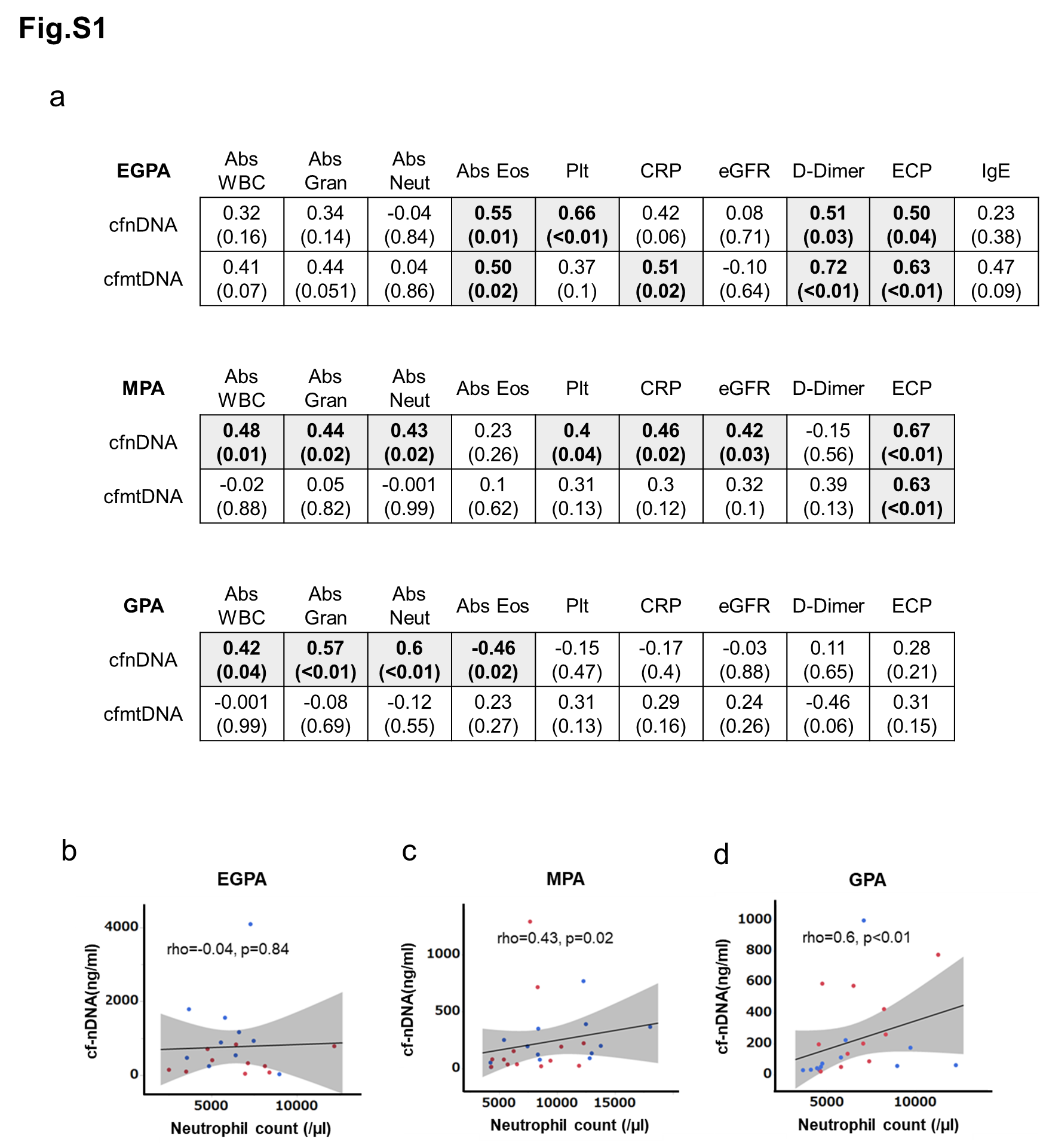


**Fig. S1** Correlations between cfDNA levels and laboratory test results in patients with AAV.

(a) Spearman’s correlation coefficients presented with *P*-values in parentheses. Areas highlighted in gray indicate significant correlations. WBC: white blood cell count, Gran: granulocyte count, Neut: neutrophil count, Eos: eosinophil count, ECP: eosinophilic cationic protein. Dot-plots of correlations between neutrophil count and serum cf-nDNA levels in patients with EGPA (b), MPA (c), and GPA (d). Blue dots: before treatment, red dots: after treatment, straight line: regression line, gray area: 95% confidence interval, EGPA: n=10, MAP: n=13, GPA: n=12, Spearman’s rank correlation coefficient.


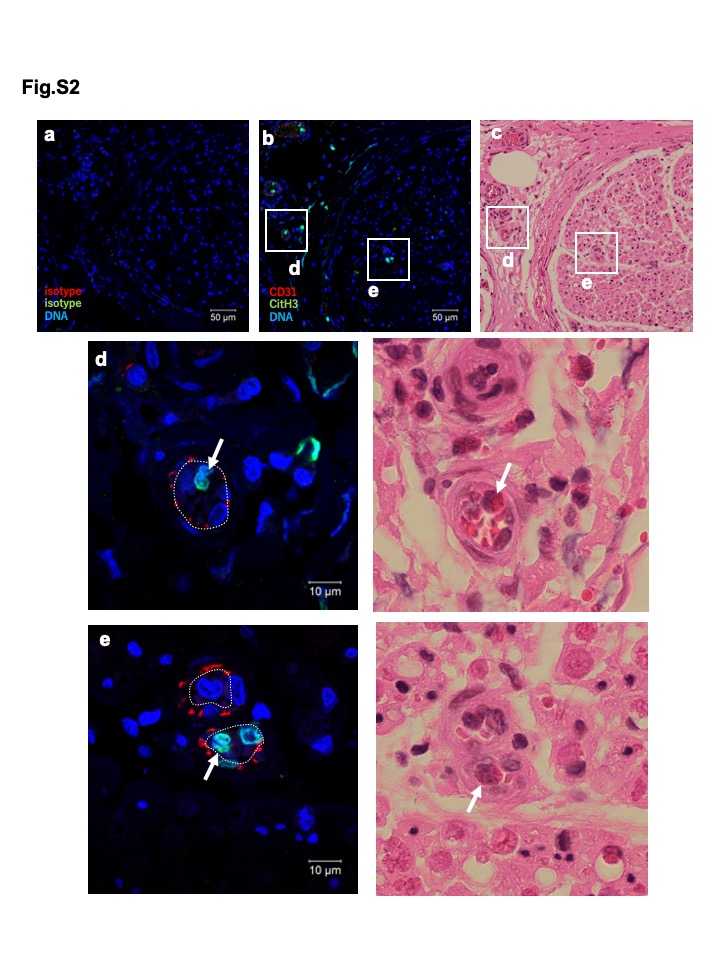
**
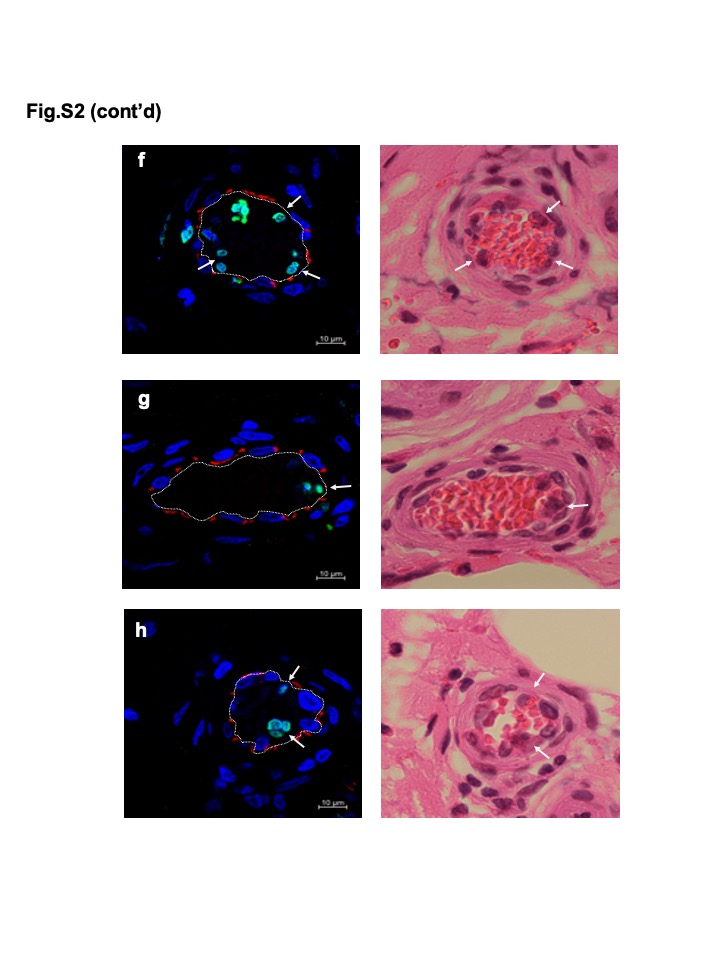
**

**Fig. S2** Citrullinated histone-positive eosinophils in blood vessel thrombi.

Sural nerve biopsy samples from patients with EGPA were assessed by immunostaining with (a) isotype-matched control antibodies and (b) specific antibodies for CD31 (red) and CitH3 (green), and (c) by H&E staining. Images (b) and (c) show identical fields from same sample section; (a) shows serial section from same sample. Images (a) and (b) obtained at same confocal microscopy setting, indicating staining specificities. (c) Magnified image of nerve trunk. (d) Extrinsic vessel and (e) intrinsic vessel, both occluded by inflammatory cells and red blood cells. (f-h) Additional representative images of thrombi from different donors. Arrows indicate CitH3-stained chromatolytic eosinophils.


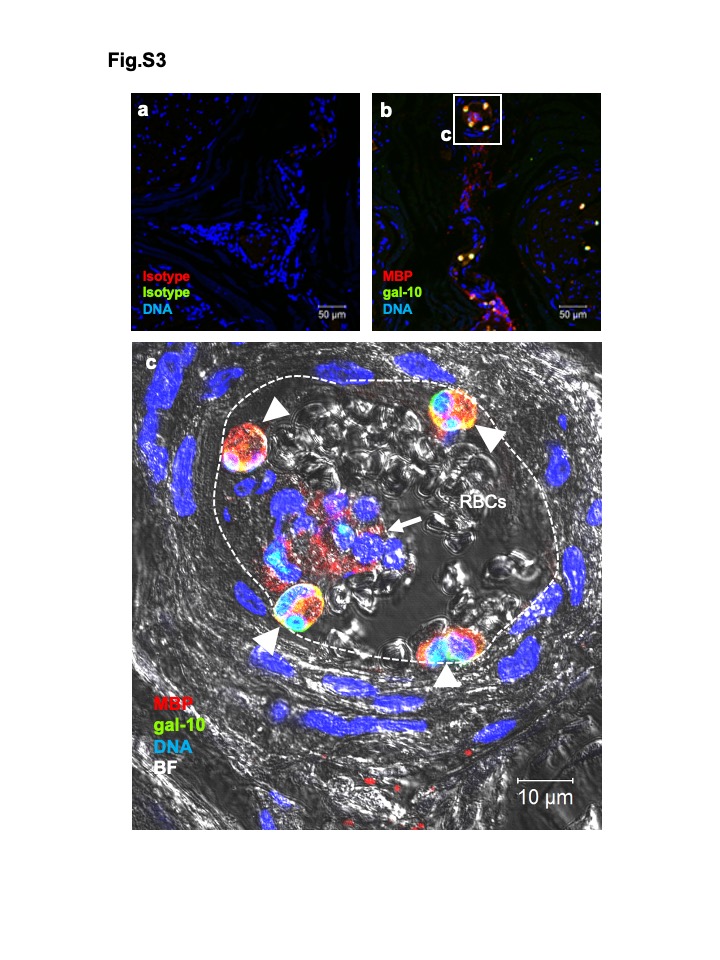


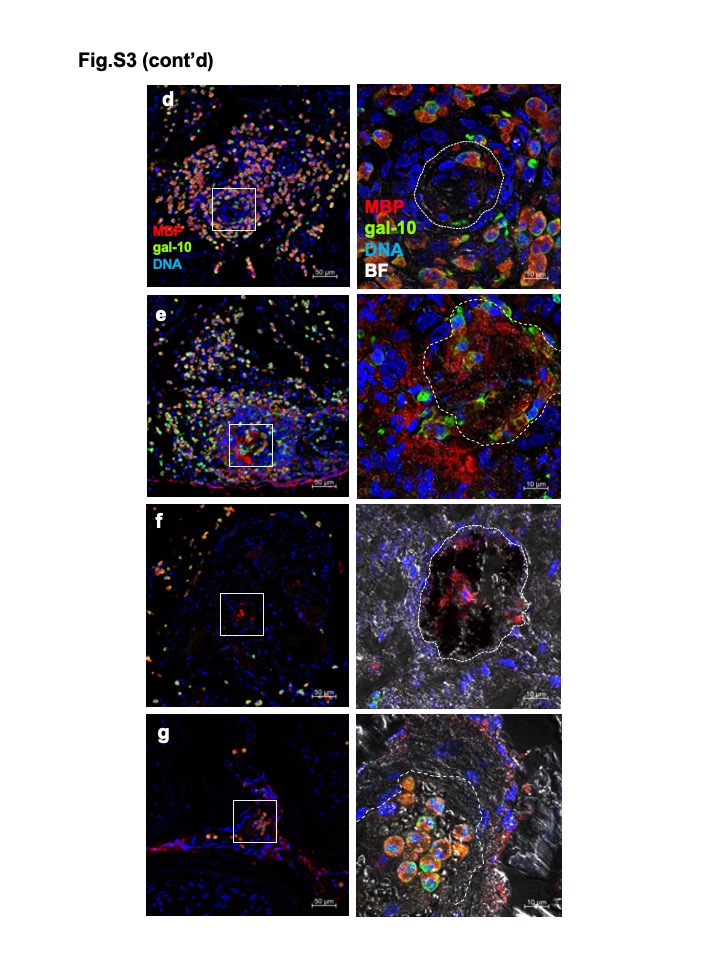


**Fig. S3** Intact and lytic eosinophils in small vessel thrombi.

Sural nerve biopsy samples from patients with EGPA were assessed by immunostaining using (a) isotype-matched control antibodies and (b) specific antibodies for MBP (red) and galectin-10 (green). Images (a) and (b) obtained at same confocal microscopy setting, indicating staining specificities. (c) Magnified image of extrinsic vessel showing intravascular thrombus with red blood cells (RBCs), intact eosinophils (arrowheads), and clustered lytic eosinophils (arrow). (d-g) Additional representative images. The cytolytic eosinophils in thrombi were present in various degree.

**
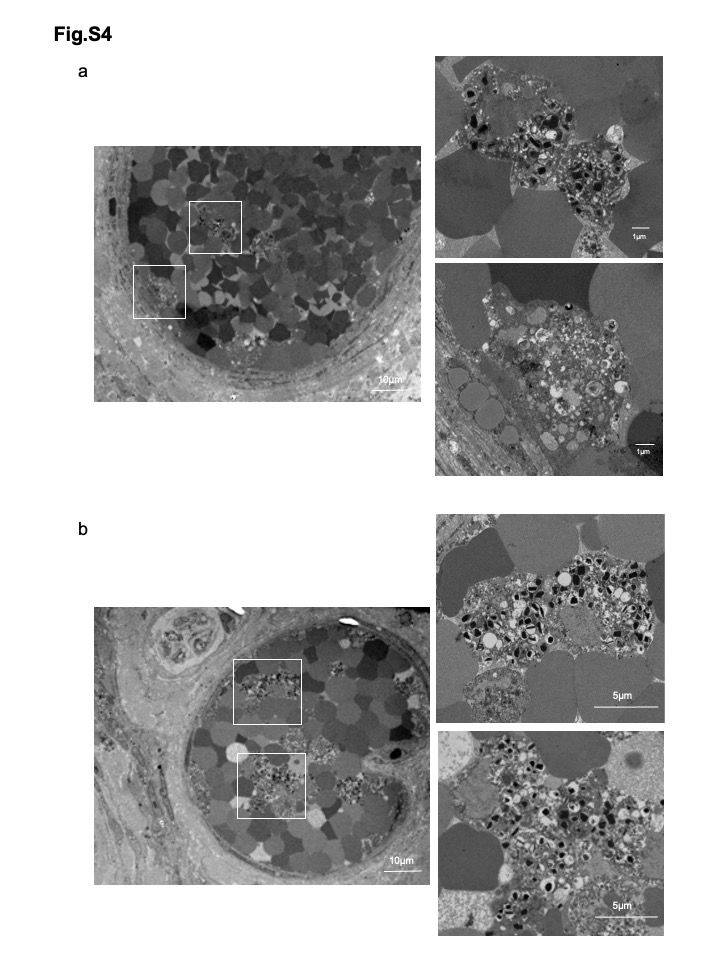
**

**Fig. S4** TEM of blood vessel thrombi in patients with EGPA.

(a, b) Additional representative TEM images are shown. Thrombus in sural nerve tissues showing lytic eosinophils with EETotic morphologies. Boxed areas in left panels are magnified in right panels.


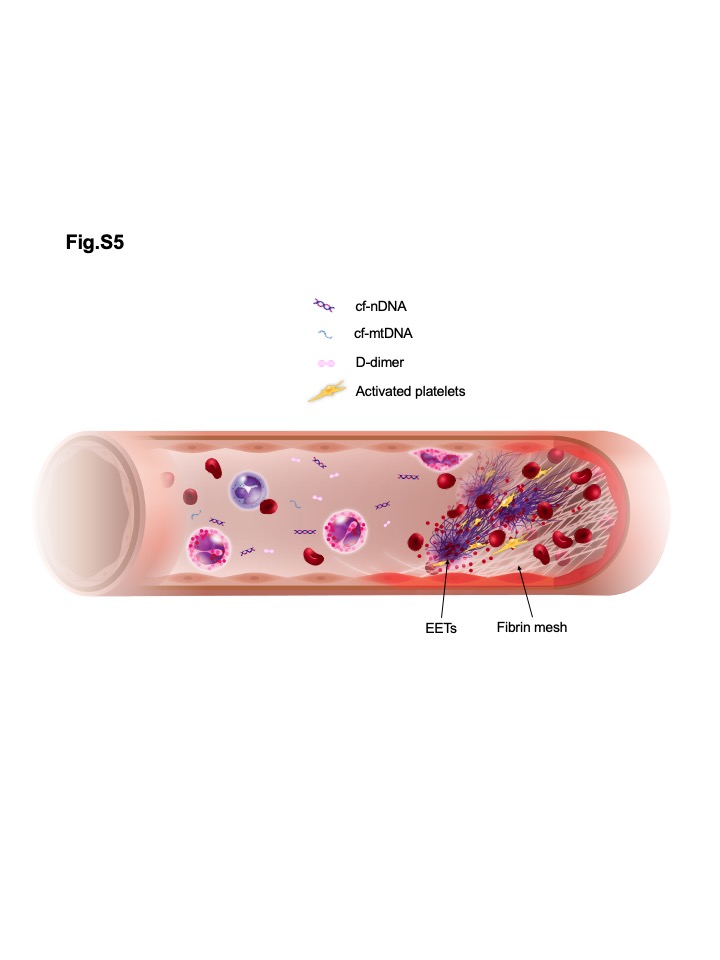


**Fig. S5** EETs/EETosis in thrombus in EGPA.

Graphic diagram showing thrombus in affected tissue in EGPA. Activated eosinophils can undergo programmed cell death via EETosis to release condensed chromatin structure EETs. EETs might provide a scaffold for platelets and promote vascular injury, leading to immunothrombosis. Increased cf-nDNA and cf-mtDNA levels might originate from cytolytic eosinophils in the thrombus.

**
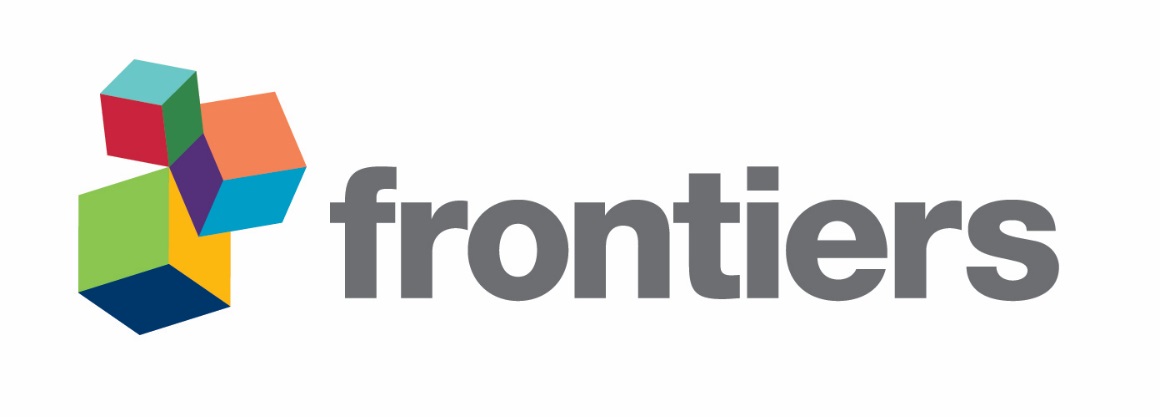
**

.
